# Anti-apoptotic effect of adipose tissue-derived stromal vascular fraction in denervated rat muscle

**DOI:** 10.1186/s13287-021-02230-y

**Published:** 2021-03-04

**Authors:** R. El-Habta, G. Andersson, P. J. Kingham, L. J. Backman

**Affiliations:** 1 Department of Integrative Medical Biology, Section for Anatomy, Umeå University, SE-901 87 Umeå, Sweden; 2 Department of Surgical and Perioperative Sciences, Section for Hand and Plastic Surgery, Umeå University, Umeå, Sweden; 3 Wallenberg Centre for Molecular Medicine, Umeå University, Umeå, Sweden; 4 Department of Community Medicine and Rehabilitation, Physiotherapy, Umeå University, Umeå, Sweden

**Keywords:** Apoptosis, Myoblast, Nerve injury, Regeneration, Skeletal muscle, SVF

## Abstract

**Background:**

Recovery of muscle function after peripheral nerve injury is often poor, and this can be attributed to muscle fiber atrophy and cell death. In the current study, we have investigated the effects of stromal vascular fraction (SVF) on muscle cell apoptosis and its potential to preserve muscle tissue following denervation.

**Methods:**

Rat gastrocnemius muscle was denervated by sciatic nerve transection. At 2 and 4 weeks after injury, muscles were examined histologically and apoptosis was measured using TUNEL assay and PCR array for a range of apoptotic genes. Additionally, an in vitro TNF-α apoptosis model was established using SVF cells co-cultured indirectly with primary rat myoblasts. Annexin V and TUNEL were used together with Western blotting to investigate the signaling pathways.

**Results:**

Denervated muscles showed significantly higher TUNEL reactivity at 2 and 4 weeks following nerve injury, and an increased expression of caspase family genes, mitochondria-related apoptotic genes, and tumor necrosis factor family genes. In cultured rat primary myoblasts, Annexin V labeling was significantly increased at 12 h after TNF-α treatment, and this was followed by a significant increase in TUNEL reactivity at 48 h. Western blotting showed that caspase-7 was activated/cleaved as well as the downstream substrate, poly (ADP-ribose) polymerase (PARP). Co-culture of myoblasts with SVF significantly reduced all these measures of apoptosis. Bax and Bcl-2 levels were not changed suggesting that the TNF-α-induced apoptosis occurred via mitochondria-independent pathways. The protective effect of SVF was also shown in vivo; injections of SVF cells into denervated muscle significantly improved the mean fiber area and diameter, as well as reduced the levels of TUNEL reactivity.

**Conclusions:**

This study provides new insights into how adipose tissue-derived cells might provide therapeutic benefits by preserving muscle tissue.

## Introduction

The incidence of peripheral nerve injuries is approximately 3% in trauma-injury patients [1]. Although some injuries may spontaneously recover, in the majority of cases, the damage to the nerve is irreversible. For these patients, surgery is the only option to restore function to the affected limb. Despite numerous advances in peripheral nerve surgery, nerve repair only results in successful reinnervation of denervated muscle when the distance required for axonal regeneration is not too great [2]. In long-term denervated muscle, where reinnvervation is delayed, functional recovery is poor due to the progressive replacement of muscle tissue by fibrous connective tissue and fat [3]. Different approaches have been used to address this issue, such as electrical stimulation, physical therapy, or cell transplantation [4–6].

Previously, Schaakxs et al. showed in an experimental animal model that injections of in vitro-stimulated adipose-derived stem cells (ASCs) into denervated muscle reduced the atrophy and enhanced hind limb functionality [7]. Furthermore, we have previously shown that denervated muscle has increased expression of muscarinic acetylcholine (ACh) receptors and that ASCs are capable of increasing the proliferation of myoblasts in vitro through paracrine secretion of ACh [8]. In this current study, we hypothesized that injections of freshly isolated adipose-tissue stromal vascular fraction (SVF) would maintain the integrity of the muscle fibers during the period of nerve regeneration. More specifically, we hypothesized that the SVF cell secretome has anti-apoptotic properties, and that combined with its ability to enhance proliferation in vitro [9], could be one-way muscle atrophy is attenuated in vivo.

SVF is a heterogenous cell population that contains multipotent ASCs, endothelial cells, smooth muscle cells, and several types of leukocytes [9, 10]. The benefits of SVF use in regenerative medicine were originally attributed to the ability of the adipose-derived mesenchymal stem cells (MSCs) to differentiate into the cells of the damaged tissues. However, the other cell types found in SVF, such as endothelial cells (and their progenitors) and pericytes, are important modulators of tissue regeneration. Thus many studies have described the pro-healing functions of SVF. For example, in one study, intra-tendinous injections of SVF in patients with Achilles tendinopathy showed significant clinical improvement in terms of pain relief and function restoration [11], and in another study, intra-articular injections in the knee of patients with osteoarthritis also reduced pain and improved cartilage thickness [12]. These studies were however unable to show that the cells actually differentiated into new tissue or whether they stimulated healing via a paracrine effect, e.g., by modulating the inflammatory response following an injury. Over the last two decades, the body of literature supporting a cell secretome-based therapy has grown exponentially. This hypothesis postulates that the administered cells themselves have trophic functions through their secretome that are important for extracellular matrix remodeling and tissue regeneration [13]. We know from our previous studies that SVF cells express a multitude of growth factors that could be beneficial in an inflammatory and muscle-atrophic environment [9], effects that might get lost with the other cell types if adipose MSCs are expanded in culture. Such factors include hepatocyte growth factor (HGF), a protein required for self-repair of injured muscle [14–16]. The anti-apoptotic nature of SVF has however not been as extensively studied as its potential to differentiate into other cell types, although it does seem promising. For example, recently, Lee et al. [17] described how SVF injections significantly decreased apoptosis and TUNEL labeling in a rat model of acute kidney injury, and Tenenhaus et al. [18] showed how necrosis after soft tissue reconstruction can be minimized using SVF injections.

In this current study, we examined the expression of apoptotic genes in an animal model of muscle denervation. Experimentally we used tumor necrosis factor alpha (TNF-α) to induce apoptosis in rat primary myoblasts in vitro, and investigated if SVF could prevent apoptosis using indirect co-culture assay. We also investigated the signaling pathways that accounted for the observed effects. Lastly, the effect of SVF injection into the denervated rat gastrocnemius muscle was determined.

## Materials and methods

### Animals and experimental design

For in vivo experiments and isolation of adipose tissue-derived stromal vascular fraction (SVF) 10–12 weeks old female Sprague-Dawley rats (Taconic Europe A/S) were used. The animal care and experimental procedures were carried out in accordance with the Directive 2010/63/EU of the European Parliament and of the Council on the protection of animals used for scientific purposes and was also approved by the Northern Swedish Committee for Ethics in Animal Experiments (No. A186-12 and A50-13). Three experimental groups (*n* = 6 rats in each group) were included: control (i.e., non-denervated), denervated (received sham-injections), and treated (received intramuscular injections of SVF), and two different time-points were examined: 2 and 4 weeks. In total, 36 rats were used in the study. Muscle denervation was only performed on one side and the contralateral side muscles served as control. Sciatic nerve transection was performed as follows. Surgery was performed using aseptic conditions under general anesthesia using isoflurane gas. Using a dorsal gluteal muscle-splitting incision, the left sciatic nerve and its branches were exposed. The sciatic nerve was carefully mobilized and transected at 5 mm proximal to the bifurcation of the tibial and common peroneal nerves. Any regenerating axons from the proximal nerve stump were blocked by use of polyethylene caps sutured to the stump with 10–0 Ethilon. After surgery, the wound was closed in layers and Finadyne (Schering-Plough Animal health 50 mg/ml) was administered post-operatively. The well-being of the rats was observed throughout the experimental period.

### Intramuscular injections

In the experimental treatment group, immediately after sciatic nerve transection and capping, the animals received one injection of 1 × 10^6^ SVF cells in a total volume of 150 μl of growth medium. The injections were made directly into the denervated gastrocnemius muscle around the point of entry of the tibial nerve. The control group received sham injections of 150 μl of normal growth medium alone. The rats received no further injections during the 4-week post-surgery period.

### Sampling and sectioning

After 2 and 4 weeks of denervation, the gastrocnemius muscle of both legs was collected, mounted on thin cardboard in OCT embedding medium (Miles Laboratories), frozen in isopropane-chilled liquid nitrogen, and then stored at − 80 °C until use. Sectioning was performed at − 22 °C using a cryostat-microtome. Series of 12 μm thick transverse sections were collected and mounted on glass slides.

### Immunohistochemistry and muscle fiber evaluation

Sections were air-dried and blocked with serum before incubation with slow type myosin heavy chain (Leica Biosystems; #NCL-MHCs; dilution 1:40) and Laminin antibody (Sigma; #L9393; dilution 1:200). Sections were incubated with primary antibody for 2 h at room temperature, and secondary antibody (Dako; #R0156; and Invitrogen; #A11029) for 60 min at room temperature in the dark. Sections were also processed for TUNEL labeling. For each muscle sample (*n* = 6 rats in each group), five images were captured of each transverse section taken at the anterior, posterior, medial, lateral, and central positions at × 40 magnification and transferred to a computer for further processing. Using Image-Pro Plus (Media Cybernetics), all fibers in each image were analyzed in respect to muscle fiber area and diameter, which corresponded to approximately 200 muscle fibers per animal. Thereafter, a mean value was calculated for each animal, and based on that, an overall mean value for each experimental group.

### TUNEL assay

To evaluate apoptosis, nuclear DNA fragmentation was measured in the muscle tissue and also in cells grown on 8-well culture slides (Falcon; #354108) using the DeadEnd Fluorometric TUNEL System (Promega; #G3250). In brief, slides were fixed in 4% paraformaldehyde in PBS, permeabilized in 0.2% Triton X-100 in PBS, equilibrated in Equilibration Buffer (provided by the manufacturer), and labeled with fluorescein-12-dUTP using Terminal Deoxynucleotidyl Transferase (TdT). Nuclei were stained using ProLong Diamond Antifade Mountant with DAPI (Invitrogen; #36962). Apoptotic cells were visualized by fluorescence microscopy. Apoptosis detection in tissue was performed similarly, with the exception of adding 20 μg/ml Proteinase K solution instead of Triton X-100 for permeabilization. TUNEL reactivity was analyzed identically as the mean muscle fiber area and diameter, i.e., all positive myonuclei were counted in each muscle sample.

### Isolation of cells and cell culturing

#### Rat primary myoblasts

Muscles from healthy female Sprague-Dawley rats (Taconic Europe A/S) were cut into small blocks (approximately 1 mm^3^) and put in a culture dish containing Dulbecco’s modified Eagle’s medium (DMEM; Thermo Fisher Scientific; #31966021), 10% fetal bovine serum (FBS; Thermo Fisher Scientific; 15140122), and 1% penicillin-streptomycin. The culture dish was incubated in a 37 °C, 5% CO_2_ incubator with media exchange every 2 days for approximately 2 weeks. When the outgrowing cells reached about 80% confluence the tissue blocks were discarded and the cells were moved to a collagen-coated dish (Thermo Fisher Scientific; #A11428-01) for 15 min to allow attachment of the rapidly adhering cells, predominantly fibroblasts. This step was repeated two more times with the medium containing the non-adherent cells. The final resulting media containing primary myoblasts was then transferred to a new flask for further culturing. Myoblasts were kept at a sub-confluent level (< 80%) to prevent the loss of myoblastic component as the cells were passaged. Phenotype and purity were confirmed by labeling for myogenin (essentially as described for muscle tissue immunohistochemistry).

#### Stromal vascular fraction (SVF) cells

SVF cells were isolated from healthy female Sprague-Dawley rats as described elsewhere [9]. In summary, fat was minced in 0.2% collagenase type I (Thermo Fisher Scientific; #17100017) in HBSS and placed in a 37 °C water bath for 60 min. The enzyme solution was neutralized using Minimum Essential Medium (MEM; Thermo Fisher Scientific; #32561029) supplemented with 10% FBS and filtered through a 70-μm cell strainer. Then, red blood cells were lysed using Ammonium-Chloride-Potassium (ACK) lysing buffer (Thermo Fisher Scientific; #A1049201) for 3 min. ACK buffer was removed by washing/centrifugation and the resulting SVF pellet was resuspended in MEM supplemented with 10% FBS and 1% penicillin-streptomycin.

### Indirect co-culture

Myoblasts were seeded in a 6-well plate (Corning; #353502) at a density of 200,000 cells per well (21,000 cells/cm^2^). In parallel, an equal amount of SVF cells (200,000 cells; 48,000 cells/cm^2^) were added in 1.0 μm PET transwell membrane inserts (Corning; #353102) and kept in a separate plate in the incubator until use. When myoblasts were 80% confluent they were starved over night by switching to a medium containing only 2% FBS. This was done to synchronize all cells to the same cell cycle phase, so that the effect of the treatment would not disappear in the cells’ own proliferation. Apoptosis was then induced by stimulating the cells with TNF-α (5–20 ng/ml). In some experiments, 20 μM of Caspase Inhibitor Z-VAD-FMK (Promega; #G7231) was added at the same time as apoptosis was induced. After 30 min of TNF-α stimulation, the transwell membrane inserts containing the SVF cells were transferred to the wells containing myoblasts, without any medium exchange. Cells were co-cultured within the TNF-a environment in 2% FBS for up to 48 h and analyzed using either caspase activity assay, Western blotting, Annexin V-, or TUNEL assay.

### Extraction of proteins from the cytosol and mitochondria

Primary rat myoblasts were cultured in T75 flasks and exposed to 10 ng/ml TNF-α for 2, 4, or 6 h. The cells were harvested using trypsin, washed once in ice cold PBS, and resuspended in 500 μl of × 0.1 HG buffer (× 10: 400 mM Tris-HCl pH 7.8; 250 mM NaCl; 50 mM M_g_Cl_2_) supplemented with Digitonin (1:1000) and Protease Inhibitors (1:200) for 15 min on ice. Using a pre-chilled Dounce tissue grinder (Kimble; #885303-0002; #885301-0002), the cells were carefully homogenized. To confirm adequate homogenization, the cell suspension was diluted 1:1 with Trypan Blue and observed under a light microscope. Once completely homogenized, the homogenate was transferred to a microcentrifuge tube and centrifuged at 700×*g* for 10 min at 4 °C. The supernatant, containing the cytosol and mitochondria, was transferred to a new microcentrifuge tube and centrifuged at 10,000x*g* for 30 min at 4 °C to pellet the mitochondria. The mitochondrial pellet was prepared for Western blotting by resuspending it in 40 μl of RIPA buffer supplemented with Protease Inhibitors (1:200). Collected fractions were diluted in Laemmli buffer (Bio-Rad; #1610747), denatured for 5 min at 95 °C, and stored at − 80 °C until use.

### Caspase activity assay

Detection of caspase 3/7 and caspase 8 activity was performed using the Caspase-Glo Assay System (Promega; #G8091; and #G8201) following the instructions provided by the manufacturer. Myoblasts were cultured in a 96-well plate (10,000 cells/well) or a 24-well plate (50,000 cells/well) when co-cultured with SVF, and stimulated with TNF-α for up to 24 h. Then, equal volume of reagent to medium was added, mixed briefly using a plate shaker, and incubated for 30 min at room temperature. Thereafter, luminescence was recorded in a plate-reading luminometer.

### Annexin V assay

Apoptosis was induced in primary rat myoblasts using 10 ng/ml TNF-α. Early apoptotic cells were identified using Annexin V-FITC Early Apoptosis Detection Kit (Cell Signaling; #6592) following the instructions provided by the manufacturer. In brief, cells were collected by centrifugation, washed in ice-cold PBS, resuspended in Annexin V binding buffer (provided by the manufacturer), and labeled with Annexin V-FITC conjugate and Propidium Iodide (PI) solution. Cells were analyzed using flow cytometry (BD LSR II).

### Quantitative reverse transcription PCR (RT-qPCR)

Muscle biopsies were homogenized in QIAzol (Qiagen; #79306) using a handheld tissue ruptor. The homogenate was placed at the benchtop for 5 min to promote dissociation of nucleoprotein complexes. Chloroform was then added to the tube (ratio of 1:5) and shaken vigorously for approximately 15 s. The homogenate was centrifuged at 18,600×*g* at 4 °C for 15 min, and the upper aqueous phase was transferred to a new tube and mixed with 1.5 vol of 100% ethanol. Total RNA was extracted using the RNeasy Mini Kit (Qiagen; #74106); cDNA was synthesized using the High Capacity cDNA Reverse Transcription kit (Applied Biosystems; #4268813); and qRT-PCR was performed with TaqMan gene expression assay (Applied Biosystems). Using the Rat Apoptosis RT^2^ Profiler PCR Array, the expression of 84 key genes involved in programmed cell death was evaluated (Qiagen; #PARN-012ZA). The amplification was performed on a ViiA 7 Real-time PCR system (Applied Biosystems). Thermal-cycling conditions were 50 °C for 2 min, 95 °C for 20 s, and 40 cycles of 95 °C for 1 s, and 60 °C for 20 s. Data were analyzed with ViiA 7 software (Applied Biosystems) and PCR Array Analysis Web Portal (Qiagen). The expression was normalized to Rplp1 levels.

### Western blotting

Myoblasts were lysed in RIPA buffer supplemented with Protease Inhibitors (Sigma-Aldrich). After approximately 30 min on ice, the cell suspension was centrifuged at 13,400×*g* for 10 min at 4 °C. Total protein was quantified using BioRad Protein Assay (Bio-Rad Laboratories; #500-0006). Samples were diluted in Laemmli buffer (Bio-Rad; #1610747), boiled for 5 min at 95 °C, and run on pre-cast polyacrylamide gels (Bio-Rad Laboratories) for 60 min at 150 V. The gels were then transferred to polyvinylidene fluoride (PVDF) membranes and run for 60 min at 100 V. Membranes were blocked in 5% bovine serum albumin (BSA; Sigma-Aldrich; #A2058) in TBST (10 mM Tris Base, 100 nM NaCl, 0.1% Tween-20) for 60 min at room temperature, and stained with antibodies against Caspase-3 (Cell Signaling; #9662), Cleaved Caspase-3 (Cell Signaling; #9664), Caspase-7 (Cell Signaling; #9492), Cleaved Caspase-7 (Cell Signaling; #9491), PARP (Cell Signaling; #9542), Cleaved PARP (Cell Signaling; #94885), Cytochrome c (Cell Signaling; #11940), TFAM (Sigma; #AV-36993), Bax (Cell Signaling; #2772), Bcl-2 (Abcam; #Ab194583), or β-actin (1:2000) (Cell Signaling; #4970) over night at 4 °C. Membranes were washed with TBST for 6 × 5 min, and incubated with either mouse or rat HRP-conjugated secondary antibodies (1:2000) (Cell Signaling; #7074 or #7076) for 60 min at room temperature. Lastly, membranes were incubated in ECL solution (GE Healthcare; #RPN2232) for 60 s and analyzed in an Odyssey Fc Dual-Mode Imaging System (LI-COR Biosciences).

### Statistical analysis

All experiments were conducted at least three times using cell preparations obtained from at least three different animals. In vivo data is based on six animals (*n* = 6) in each of the three experimental groups and for both time-points (2 and 4 weeks). Results are presented as mean ± standard deviation. Statistical analysis was performed using GraphPad Prism 7. Statistical differences were analyzed using one-way analysis of variance (ANOVA) with post hoc test (Bonferroni correction) or Student’s *t* test. *P* < 0.05 was considered statistically significant. For the Rat Apoptosis RT^2^ Profiler PCR Array, RNA from all animals within each experimental group at the 2-week time-point were combined into one sample. No statistical significance could therefore be calculated.

## Results

### Increased apoptosis in denervated muscles

**Fig. 1:**
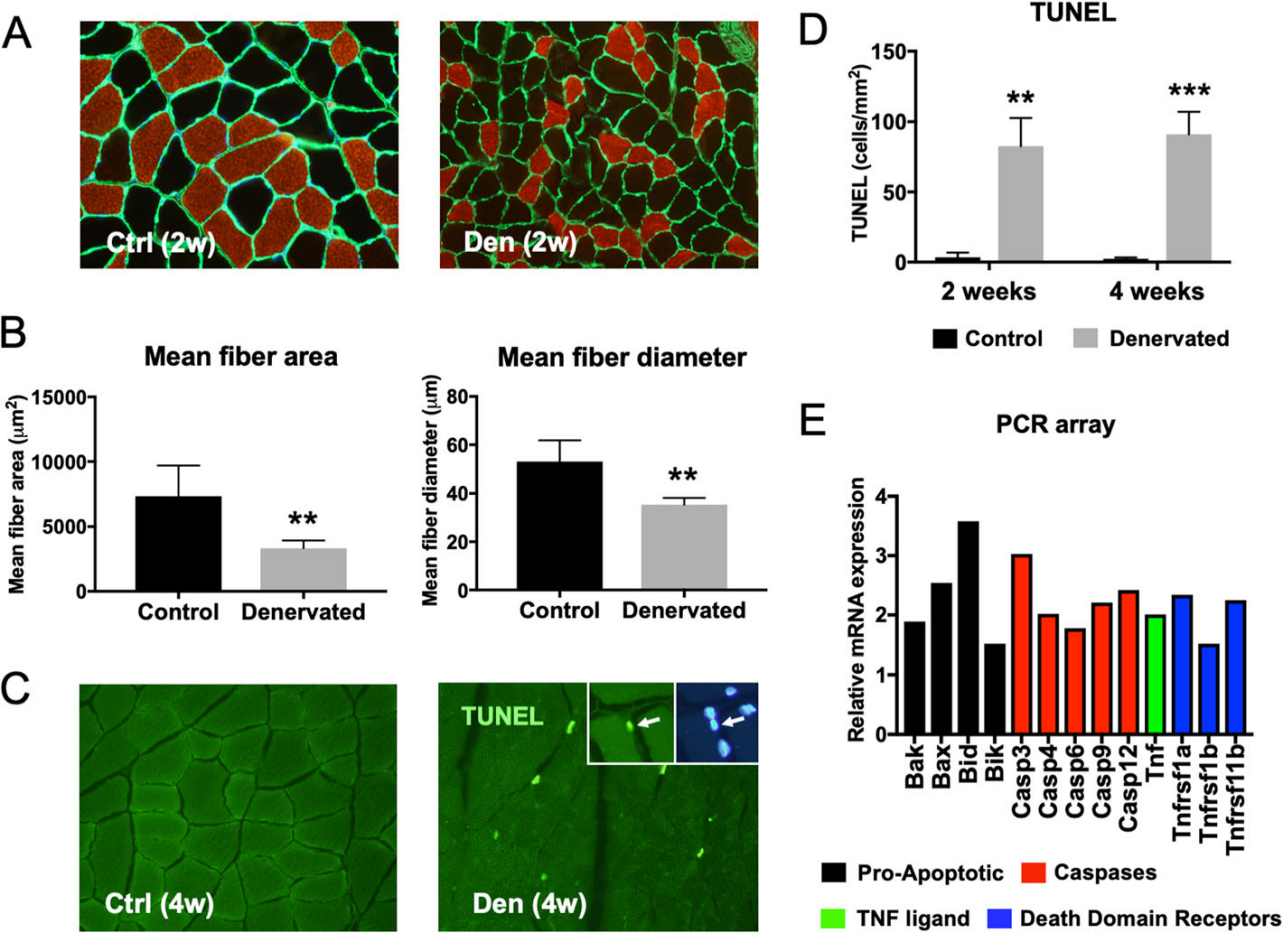
Apoptosis-related signaling in denervated rat gastrocnemius muscle. **a** Histological analyses of rat gastrocnemius muscles after 2 weeks of denervation (den). The non-denervated, contralateral side was used as a control (ctrl). Slow type muscle fibers were labeled with anti-Myosin heavy chain 1 antibody (red), and anti-Laminin antibody (green). × 40 magnification. **b** Comparison of the mean fiber area and diameter of slow type fibers in denervated and control muscle. **c** Labeling of DNA fragmentation using TUNEL assay in samples denervated for 4 weeks. × 40 magnification. Insert shows a high magnification TUNEL-positive myonucleus co-labeled with DAPI. **d** Quantification of the number of TUNEL-positive cells in denervated and control muscle after 2 and 4 weeks of denervation. **e** Relative expression of key genes involved in programmed cell death in 2-week denervated muscle compared to control. For all experiments, *n* = 6. Statistical significance was calculated using Student’s *t* test. **P < 0.01, and ***P < 0.001

Denervated muscles showed significantly reduced mean fiber area and diameter at 2 weeks after nerve injury compared to control muscles (Fig. 1a, b). In these fibers as well as in fibers denervated for 4 weeks, there was a significant increase in TUNEL reactivity (Fig. 1c, d). Additionally, denervated muscles had an increased expression of caspase family genes, mitochondria-related apoptotic genes, and tumor necrosis factor (TNF) family genes (Fig. 1e).

### TNF-α induces apoptosis in rat primary myoblasts

**Fig. 2:**
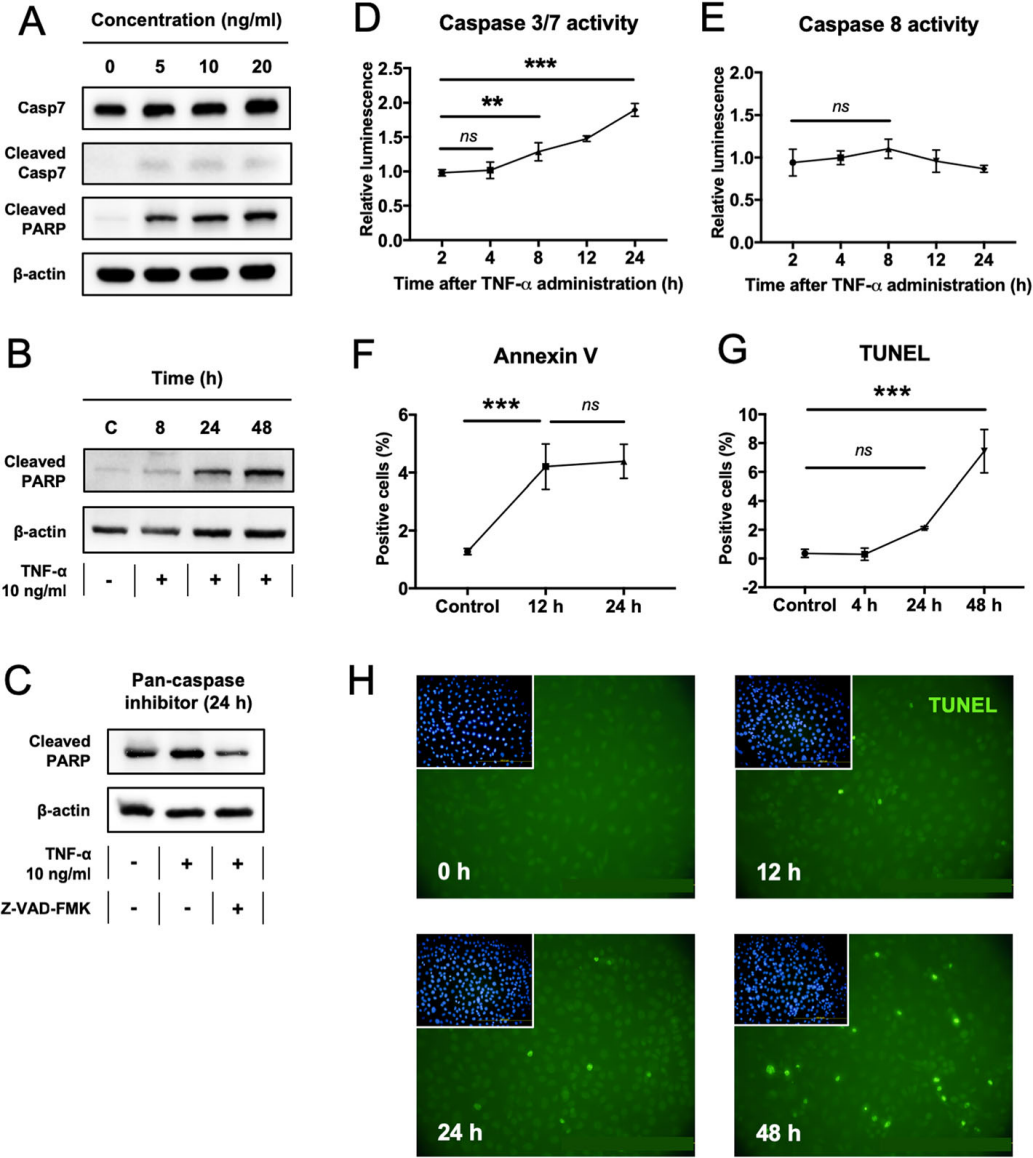
TNF-α-induced apoptosis in primary rat myoblasts. **a** Western blot showing the activation/cleavage of caspase 7 and PARP in myoblasts exposed to different concentrations of TNF-α (5–20 ng/ml). **b** The cleavage of PARP at different time-points (0–48 h). **c** The effects of a pan-caspase inhibitor (Z-VAD-FMK) on the cleavage of PARP. **d**, **e** Caspase 3/7 activity (**d**) and caspase 8 activity (**e**) in myoblasts after treatment with 10 ng/ml TNF-α for up to 24 h. **f** The percentage of Annexin V-positive cells (early apoptotic cells) after treatment with 10 ng/ml TNF-α for up to 24 h. Data is from flow cytometry experiments. *n* = 3. **g** The percentage of TUNEL-positive cells (late apoptotic cells) after treatment with 10 ng/ml TNF-α for up to 48 h. *n* = 6. **h** TUNEL labeling time course up to 48 h. DAPI staining shown in the upper left corner. × 20 magnification. Statistical significance was calculated using one-way ANOVA with post hoc test (Bonferroni correction). **P < 0.01, and ***P < 0.001. *ns* = not significant

Rat primary myoblasts were treated with 0, 5, 10, and 20 ng/ml TNF-α. Western blotting indicated that effector caspase-7 was activated/cleaved in response to TNF-α treatment, as was the known downstream substrate poly ADP-ribose polymerase (PARP) (Fig. 2a, b). The cleavage of PARP was both TNF-α dose (Fig. 2a) and time-dependent (Fig. 2b). The addition of a pan-caspase inhibitor (Z-VAD-FMK) indicated that the cleavage of PARP was caspase-dependent (Fig. 2c). Caspase 3/7 activity was significantly upregulated following TNF-a treatment (Fig. 2d) but did not affect the activity of caspase 8 significantly (Fig. 2e). Annexin V labeling was significantly increased at 12 h after TNF-α treatment (Fig. 2f) and this was followed by a significant increase in TUNEL labeling at 48 h (Fig. 2g, h).

### Anti-apoptotic effect of SVF on TNF-α-induced apoptosis

**Fig. 3:**
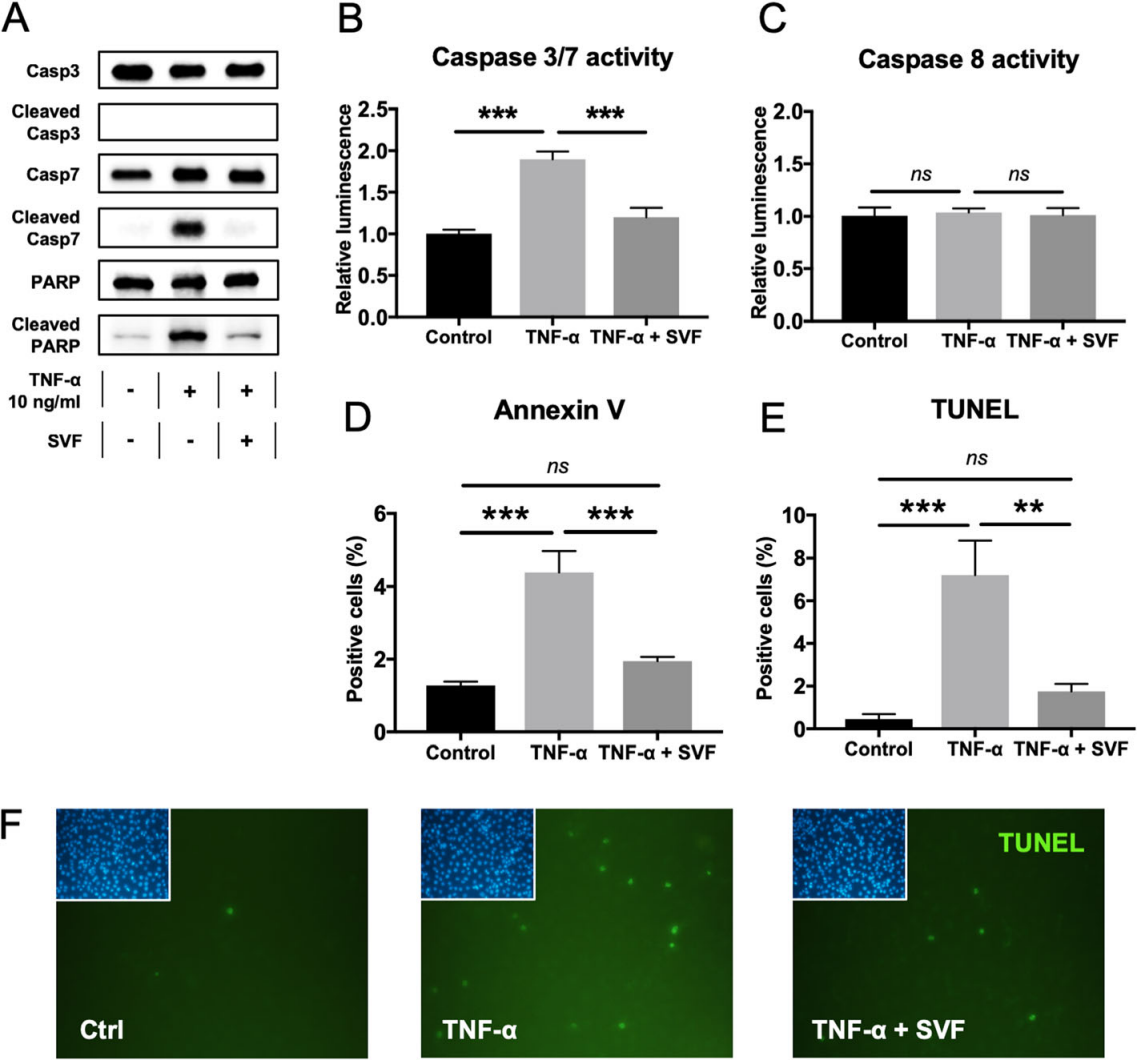
Anti-apoptotic effects of the SVF cell secretome on TNF-α-induced apoptosis. **a** Western blot showing the effects of SVF on the activation of caspase-3 and caspase-7 as well as the cleavage of PARP in myoblasts treated with 10 ng/ml TNF-α for 24 h. **b**, **c** Caspase 3/7 activity (**b**) and caspase 8 activity (**c**) in TNF-α treated myoblasts (10 ng/ml) after 24 h of indirect co-culture with SVF. **d** The percentage of Annexin-V positive myoblasts in SVF co-cultures after 24 h of TNF-α treatment (10 ng/ml). **e** The percentage of TUNEL-positive myoblasts in SVF co-cultures after 48 h of TNF-α treatment (10 ng/ml). *n* = 6. **f** TUNEL labeling in myoblasts indirectly co-cultured with SVF cells. *n* = 3. DAPI staining shown in the upper left corner. × 20 magnification. Statistical significance was calculated using one-way ANOVA with post hoc test (Bonferroni correction). **P < 0.01, ***P < 0.001. *ns* = not significant

Indirect co-culture of SVF and TNF-α-treated myoblasts significantly reduced all measures of apoptosis, including cleavage of PARP (Fig. 3a), and caspase 3/7 activity (Fig. 3b). SVF did not affect the activity of caspase 8 (Fig. 3c), but significantly reduced Annexin V- and TUNEL-reactivity (Fig. 3d–f).

### TNF-α-induced apoptosis is mitochondria-independent in vitro

**Fig. 4:**
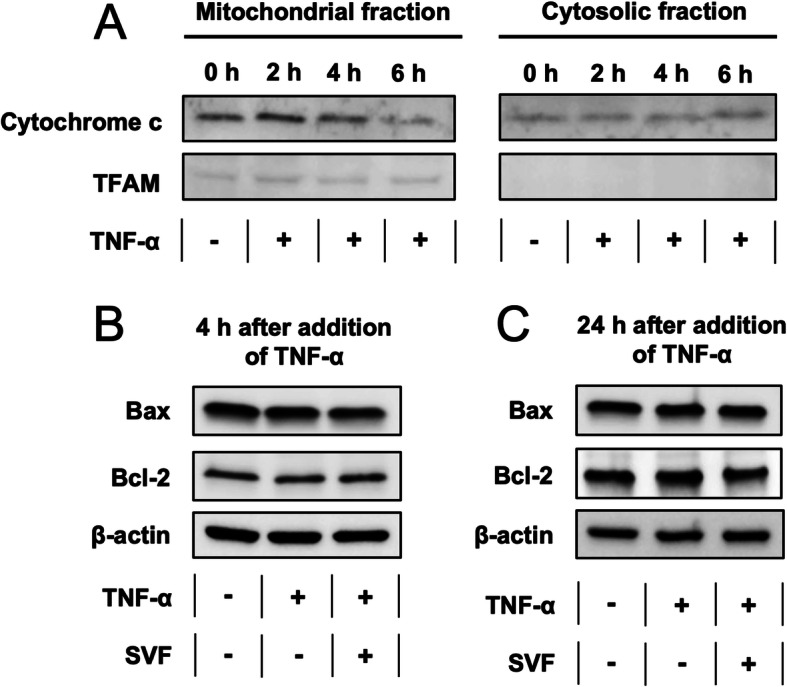
TNF-α-induced apoptosis is mitochondria-independent in vitro. **a** Cytochrome c levels in the mitochondria (left) and the cytosol (right) of myoblasts at different time points (0–6 h) after TNF-α treatment (10 ng/ml). TFAM was used as a housekeeping gene for the mitochondria. **b** The expression of mitochondria-related proteins Bax (pro-apoptotic) and Bcl-2 (anti-apoptotic) after 4 h of TNF-α treatment. **c** The expression of Bax and Bcl-2 protein after 24 h of TNF-α treatment

Western blotting showed no clear evidence of cytochrome c release after TNF-α treatment (Fig. 4a). Bax and Bcl-2 levels were equally unaffected (Fig. 4b) suggesting that the TNF-α induced apoptosis occurred via mitochondria-independent pathways. Bax translocation to the mitochondrial membrane was not observed using immuno-labeling (data not shown).

### Intramuscular injections of SVF decrease muscle apoptosis and related signaling

**Fig. 5:**
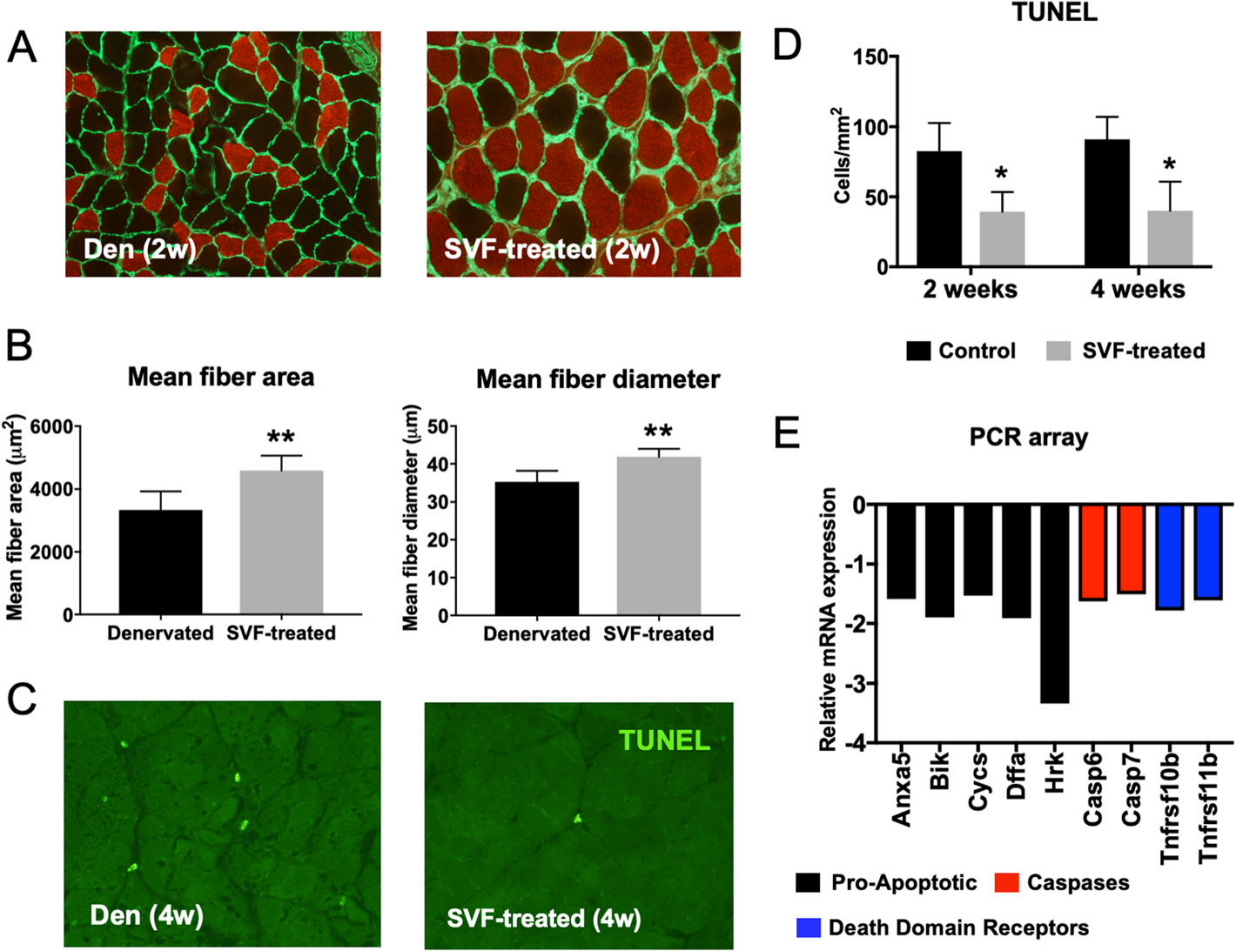
Intramuscular injections with SVF have an anti-apoptotic effect on denervated muscle. **a** Slow type fiber immunohistochemistry of denervated rat gastrocnemius muscle treated with SVF for 2 weeks. × 40 magnification. **b** Comparison of the mean muscle fiber area and diameter of denervated muscle and SVF-treated muscle. **c** TUNEL labeling in denervated muscle and in SVF-treated muscle. 40x magnification. **d** Number of TUNEL-positive cells, based on the data in **c**. **e** Expression of apoptosis-related genes in SVF-treated muscle compared to denervated muscle. *n* = 6. Statistical significance was calculated using Student’s *t* test. *P < 0.05 and **P < 0.01

The protective effect of SVF was also shown in vivo; injections of SVF cells into denervated muscle significantly improved the mean fiber area and diameter (Fig. 5a, b) and reduced the levels of TUNEL-positive myonuclei compared to sham-injected control muscle (Fig. 5c, d). Additionally, the expression of Annexin V, caspase family genes, and TNF-α receptor genes was reduced in response to SVF treatment (Fig. 5e).

## Discussion

In this study, we investigated the apoptotic events that occur in rat gastrocnemius muscle after a sciatic nerve lesion. At 2 weeks post denervation, we observed a significantly smaller mean fiber area and diameter in denervated muscle compared to control muscle, and this was followed by an increased fragmentation of DNA at 4 weeks post denervation. During the time of denervation, there was an increased expression of genes associated with apoptosis, including caspases, cytokines, and death domain receptors. This is consistent with other studies showing that apoptosis contributes to muscle mass loss during complete or partial denervation, both in patients with neuromuscular disorders [19–21] and in experimental denervation animal models [22–24]. Specific mechanisms, however, remain to be elucidated.

In denervated muscles, we observed an increased mRNA expression of tumor necrosis factor alpha (TNF-α) and the receptors TNF receptor 1 and TNF receptor 2 (TNFR1 and TNFR2, respectively). TNF-α is a cytokine that is released during various inflammatory diseases and is primarily produced by activated macrophages [25]. It is also highly expressed in injured myofibers, lymphocytes, fibroblasts, and mast cells [26]. In order to evaluate the anti-apoptotic potential of the adipose tissue-derived stromal vascular fraction (SVF), we used TNF-α as a model to induce apoptosis in myoblasts in vitro. The initial step in TNF-α signaling involves the binding of TNF-α to its receptors. Multiple studies have revealed that TNFR1 initiates the majority of TNF’s biological activities [27], such as apoptosis, whereas TNFR2 is mainly expressed by immune cells and endothelial cells [28]. Upon binding of TNF-α to TNFR1, a series of intracellular events are triggered that ultimately lead to the activation of effector caspases, NF-κB, or JNK signaling pathways [27, 28]. In our study, TNF-α treated myoblasts showed a distinct elevation of effector caspase-7, but there was no change in the expression of effector caspase-3, as evidenced by Western blotting. In denervated muscle, the results were the opposite: caspase-3 was upregulated, but there was no change in the expression of caspase-7. This could be explained by the fact that we looked at protein expression in vitro, and at mRNA expression in vivo, but it could also indicate a difference in signaling mechanisms, or depend on the time scale (hours in in vitro experiments, weeks in in vivo experiments).

Based on mRNA expression, we had indications of mitochondria-associated signaling in denervated muscle. We therefore investigated this further in myoblasts in vitro. From our Western blotting experiments, we could not detect any differences in the expression of key apoptotic factors such as Bax (pro-apoptotic) and Bcl-2 (anti-apoptotic), suggesting that TNF-α signaling was mitochondria-independent. The absence of cytochrome c in the cytosol further confirmed this conclusion. We also examined the expression of capase-8, which is involved in the extrinsic pathway and can activate caspase-7, but the expression remained unchanged after TNF-α administration. Thus, the upstream signaling mechanism that activates caspase-7 after TNF-α-administration in vitro still remains unidentified.

We know from previous studies that the SVF secretome is rich in growth factors which can enhance cell proliferation via activation of MAPK signaling, e.g., via HGF secretion [9]. MAPK signaling can also inhibit apoptosis by inhibiting pro-apoptotic factors such as Bim and Bad (via ERK1/2) and promote survival via anti-apoptotic factors such as Bcl-2 and Bcl-xL. However, since the TNF-α signaling was in this study mitochondria-independent, we suggest that the inhibition must affect proteins of the extrinsic pathway or at the receptor level. One possible mechanism is competitive binding and inhibition of TNFR1 and/or TNFR2. For example, HGF can inhibit Fas-mediated apoptosis via sequestration of Fas and c-Met on cell surfaces [29]. Perhaps a similar mechanism can be described for TNFR1 and TNFR2. It is also possible that inhibition of TNFR2 can block signals that are transduced via TNFR1 and vice versa, as there is evidence that the two receptors transduce their signals cooperatively [30, 31]. In our experiments, however, such mechanisms are debatable since TNF-α was added approximately 30 min before the addition of SVF.

When SVF was included in our experiments all measures of apoptosis, including TUNEL reactivity, were significantly improved, i.e., resulted in reduced apoptosis, both in vitro and in vivo. It should however be noted that SVF injections were only effective in reducing slow fiber atrophy. Since TUNEL reactivity was likely coupled to effector caspase activity in vitro, we speculate that the reduction in myofiber area and diameter after denervation is a consequence of apoptosis. Looking at other studies, it seems however that the degree of TUNEL reactivity in muscle can vary a lot. In one study, only a small fraction of cells with abnormal morphology had DNA breaks at 2 months post denervation [22], and in another study, TUNEL reactivity was absent in rodent hindlimb muscle following 30 weeks of denervation [32]. In our experiments, TUNEL reactivity in vivo was relatively low compared to the reduction in mean fiber area in denervated muscles. This can be explained by the fact that TUNEL represents a snapshot of the apoptotic level and can vary a lot with time. Interestingly, TUNEL reactivity is sometimes found exclusively in myofibers undergoing the process of regeneration and DNA repair [23, 32–34]. Thus, DNA breaks may have some unidentified physiologic function in coordinating the remodeling of myofibers in response to denervation.

In conclusion, TNF-α might not be the only candidate needed for recreating the events that occur in vivo following denervation. However, with our in vitro experimentation using TNF-α we could demonstrate that the SVF secretome is very potent in inhibiting apoptosis in myoblasts by blocking the activation of caspase-7 and the fragmentation of DNA, which we believe contributes to the reduced muscle fiber size in vivo. With the current data, the involvement of the mitochondria in vivo cannot be excluded, especially since there is an upregulation of mitochondria-related apoptotic markers in denervated muscle, and a down-regulation of Bik and cytochrome c in SVF-treated muscle. Also, other studies have reported mitochondria-associated apoptotic signaling in denervated rat muscle [35–37]. Nonetheless, injections of SVF into denervated muscle is a promising strategy for the treatment of denervated tissue.

## Conclusions

This study provide new insights into how adipose tissue-derived cells might provide therapeutic benefits for nerve-injured patients by preserving muscle tissue. Although the exact mechanisms of how SVF propagates its effect in vivo is unknown, we believe that paracrine secretion of mitogenic and anti-apoptotic growth factors, such as HGF, play an important role based on our in vitro co-culture model. We now want to study whether these effects can be attributed to one specific cell type, e.g. ASCs, or if it is the combined result of many cell types. Based on our findings, we believe that using the SVF in its entirety is the best choice for any cell-based therapy of denervated tissue at the moment. Cell-cell interactions, or differentiation of SVF into muscle [38], could also play a role; this however needs further study.

## Data Availability

The datasets generated and analyzed during the current study are available from the corresponding author on reasonable request.

## Abbreviations

ASC: Adipose tissue-derived stem cell
HGF: Hepatocyte growth factor
PARP: Poly ADP-ribose polymerase
RT-qPCR: Quantitative reverse transcription PCR
SVF: Stromal vascular fraction
TNF-α: Tumor necrosis factor alpha
TUNEL: Terminal deoxynucleotidyl transferase dUTP nick end labeling
